# Time of Day and its Association with Risk of Death and Chance of Discharge in Critically Ill Patients: A Retrospective Study

**DOI:** 10.1038/s41598-019-48947-y

**Published:** 2019-08-29

**Authors:** Paul Zajic, Peter Bauer, Andrew Rhodes, Rui Moreno, Tobias Fellinger, Barbara Metnitz, Martin Posch, Philipp G. H. Metnitz

**Affiliations:** 1 Div. of General Anaesthesiology, Emergency- and Intensive Care Medicine Medical University of Graz, Graz, Austria; 2 Centre for Medical Statistics, Informatics, and Intelligent Systems, Medical University of Vienna, Vienna, Austria; 3 St George’s University Hospitals NHS Foundation Trust, St George’s University of London, London, United Kingdom; 4 Unidade de Cuidados Intensivos Neurocríticos, Centro Hospitalar de Lisboa Central, Lisbon, Portugal; 5 Austrian Centre for Documentation and Quality Assurance in Intensive Care, Vienna, Austria

**Keywords:** Health services, Outcomes research

## Abstract

Outcomes following admission to intensive care units (ICU) may vary with time and day. This study investigated associations between time of day and risk of ICU mortality and chance of ICU discharge in acute ICU admissions. Adult patients (age ≥ 18 years) who were admitted to ICUs participating in the Austrian intensive care database due to medical or surgical urgencies and emergencies between January 2012 and December 2016 were included in this retrospective study. Readmissions were excluded. Statistical analysis was conducted using the Fine-and-Gray proportional subdistribution hazards model concerning ICU mortality and ICU discharge within 30 days adjusted for SAPS 3 score. 110,628 admissions were analysed. ICU admission during late night and early morning was associated with increased hazards for ICU mortality; HR: 1.17; 95% CI: 1.08–1.28 for 00:00–03:59, HR: 1.16; 95% CI: 1.05–1.29 for 04:00–07:59. Risk of death in the ICU decreased over the day; lowest HR: 0.475, 95% CI: 0.432–0.522 for 00:00–03:59. Hazards for discharge from the ICU dropped sharply after 16:00; lowest HR: 0.024; 95% CI: 0.019–0.029 for 00:00–03:59. We conclude that there are “time effects” in ICUs. These findings may spark further quality improvement efforts.

## Introduction

Medical urgencies and emergencies may occur at any time. Health care in general and critical care medicine especially are therefore required 365 days of a year, 24 hours per day. Case mix and severity of illness, however, vary markedly across the day. Paired with possible biological variability during the course of the day^1^ these aspects can lead to variation in outcomes for critically ill patients. Availability of staff and resources, processes and schedules and therefore provision of care may also vary over the course of the day and the week.

Several previously published studies have sought to identify possible effects of “off-hours” admission to both health care facilities in general and intensive care units (ICU). This existing body of evidence, however, does not allow for definitive conclusions on the subject, as these studies lack a common methodological approach and present conflicting results^2–10^. While the largest studies to date identified significant “off-hour” effects^11–13^, another recent study^14^ and a systematic review and meta-analysis failed to do so^15^. Comparability of available studies is limited by inconsistent definitions of “off-hours”, which is in turn are inevitable due to differences in rosters and shift patterns in different health care systems. Simple dichotomisation of time of day into “work-hours” and “off-hours” may not be valid for interprofessional intensive care teams in their entirety.

Some research groups, including our own^16^, focused their efforts on the so-called “weekend effect”, so only a limited amount of studies have assessed changes in outcomes according to the time of day^15^. Moreover, the available evidence focuses mainly on a possible association between admission time and outcomes. While highly relevant, these postulated associations may not be the only time-related effects in critically ill patients. It seems plausible that decision making and disposition strategies may be influenced by time of day as well, which could possibly influence outcomes. Extrapolation of findings from studies on “weekend effects” or “off-hour effects” in general supports this assertion^16^.

## Aim

This study aims to investigate, whether time of day is associated with variation in the risk of ICU mortality and chance of ICU discharge in patients admitted to critical care for medical or surgical emergencies. Since both the time of admission to the intensive care unit and the time of the events in question (death in the ICU, discharge from the ICU) are of interest, both timepoints are investigated simultaneously.

## Methods

### Data source

This study was conducted using data collected prospectively by the Austrian Centre for Documentation and Quality Assurance in Intensive Care (ASDI). As a not-for-profit organisation, ASDI set up and maintains a multicentre database on anonymised data on patients admitted to participating intensive care units in Austria. The dataset’s contents were described in detail previously^17^.

In short, the registry documents: sociodemographic data (age, sex, chronic conditions, etc.), reasons for ICU admission (recorded according to a predefined list of diagnoses^18^), severity of illness (measured by the Simplified Acute Physiology Score 3 (SAPS 3, used since 2012))^19,20^, level of provided care (measured by the Simplified Therapeutic Intervention Scoring System (TISS-28)^21^), length of ICU and hospital stay, and outcome data (survival status at ICU and hospital discharge). Reporting of these core data is required by national law, which guarantees dataset completeness. The need for informed consent was waived by the institutional review board since no interventions were performed.

Differences in work schedules and rosters factored into the decision-making process on how to model time-related variables in our model: Austrian hospital physicians work primarily during regular work hours. During off-hours, physician presence is guaranteed by on-call physicians on site. As a result, the number of physicians during regular work hours is higher than during off-hours. The minimum ratio of nursing staff to critically ill patients is regulated by law, the number of nursing personnel present in intensive care units is therefore relatively stable. Nursing staff usually work full shifts, ranging from six to twelve hours.

### Patient selection

All adult patients (age 18 years and above) admitted to participating Austrian ICUs due to medical reasons or following emergency surgery between January 1, 2012, and December 31, 2016 were included in this study. Scheduled admissions to ICU were not included. Patients, in whom data on age, date and time of admission and outcomes were missing, were excluded from the analysis. In patients readmitted to the ICU during an ongoing hospital stay, only the first admission was included.

### Statistical analysis

Basic patient demographics, severity of illness scores, length of stay and outcome data were analysed and presented using usual methods of descriptive statistics. Data are generally given as median and interquartile ranges (IQR) or absolute number (n) and percentage (%) unless specified otherwise.

This study’s main analysis was conducted using a model of proportional subdistribution hazards as proposed by Fine and Gray^22^. Events of interest in this competing risk setting were ICU mortality and ICU discharge within 30 days. Event times of patients admitted to ICUs for more than 30 days were censored in accordance with previous studies^14,16^. Analyses were conducted according to ref.^23^ with R version 3.4.2 and the package survival version 2.41–3. The model was built around an a priori defined variable list, which aimed to model both outcomes of interest and possible confounders. Only variables mandatorily documented in the registry were included to ensure model integrity.

Time-related factors – time of ICU admission and time of events – were represented as categorical variables of four hours each to allow for both sufficient granularity and model simplicity. “08:00–11:59” was chosen as the reference category for these variables. The impact of time on the hazards of events (either death in the ICU or discharge from the ICU) was modelled by a time-dependent variable.

Since so-called “weekend effects” and relatively homogenous outcomes during the week were already demonstrated in a previous study, day type of ICU admission and event (death or discharge) were dichotomised as “work day” or “non-working day” to simplify the model. “Work days” were defined as Monday to Friday, excluding public holidays. 08:00 was used as each day’s beginning to adequately represent usual shift patterns in intensive care units. Saturdays, Sundays and public holidays were categorised as “non-working days”. Day type of events was modelled as a time-dependent variable. “Work day” was chosen as the reference value for this variable.

To analyse whether possible “time effects” are evenly distributed all over the week, interaction terms between the admission time and the admission day type as well as between the event time and the event day type were incorporated into the model.

The following additional variables were used to adjust for severity of illness and system-inherent influence factors: SAPS 3 score (per 10 points) as a comprehensive measure of severity of both acute and chronic illness, type of admission (“medical” or “non-scheduled surgery”, as outlined in the SAPS 3 variable definitions^19^), to represent adjust for the need for surgery; year of admission; month of admission. Robust standard errors were calculated clustered by ICU to adjust for correlation of outcomes within centres.

Scaled Schoenfeld residuals were calculated to assess goodness of fit. Predictive power was assessed using C-statistics^24^.

Multiple sensitivity analyses were performed. The main model was applied to extended cohorts: main cohort including patients after scheduled admissions (such as major elective surgery), main cohort including readmissions. The main model described above was used with different definitions of non-working days (i.e. not accounting for holidays), beginnings and endings of days (i.e. 00:00–23:59 instead of 08:00–07:59). Based on Schoenfeld residual patterns, a further sensitivity analysis was conducted: an additional interaction between time of day of admission and time of day of event was added to the ICU discharge model in order to assess its impact on main results. Cox proportional hazards models fitted for cause-specific hazards in a competing risk setting were used to repeat the main analysis on the same patient cohort.

## Results

**Figure 1:**
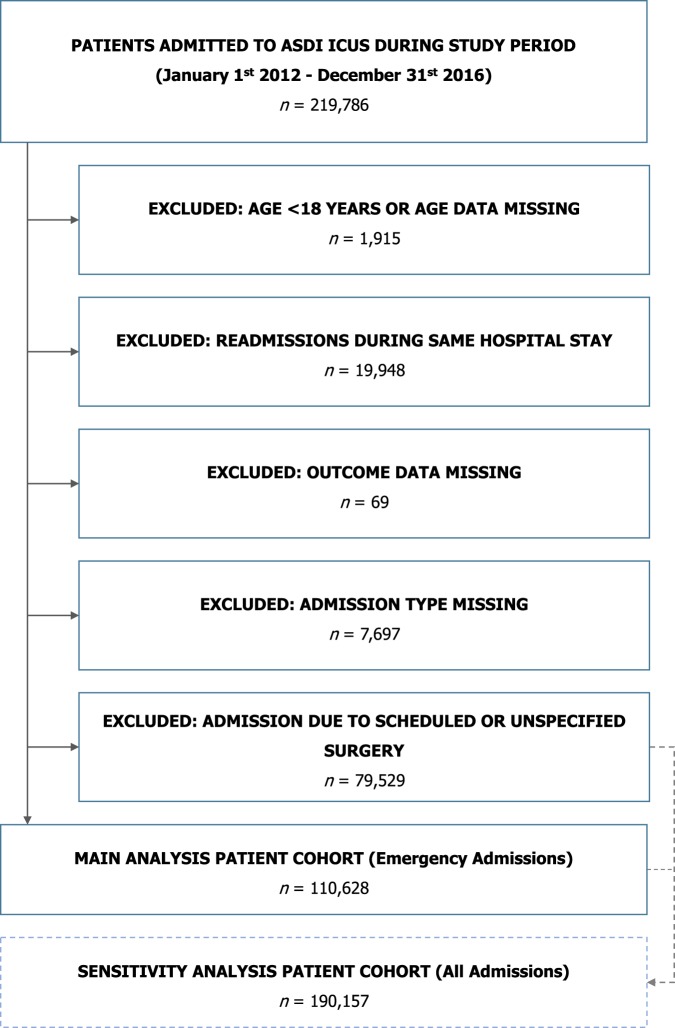
Study flow chart.

The patient cohort building process was described in Fig. 1. 219,786 patient admissions were documented in the ASDI data base during the observation period. Applying the previously described exclusion criteria yielded a total of 110,628 patient data sets from 117 ICUs to be analysed in the main analysis. The addition of patients admitted after scheduled surgery led to a cohort of 190,157 data sets available for sensitivity analyses.

Baseline patient demographics were listed in Table 1. Patients in the main analysis cohort in general were 69 (54–78) years old and predominantly male (57.9%). The majority of patients were admitted to intensive care due to medical reasons (72.9%) and remained in the ICU for a median of 3 (2–7) days. Median SAPS 3 score was 51 (43–63). 14,688 (13.3%) patients died during their ICU stay.

**Table 1:** Baseline patient demographics for the main analysis cohort overall and stratified by admission time intervals.

	Overall	00:00–03:59	04:00–07:59	08:00–11:59	12:00–15:59	16:00–19:59	20:00–23:59
*n*	110628	8897	6294	24462	30066	22882	18027
Age [years] (median, IQR)	69 (54–78)	65 (49–76)	66 (51–77)	70 (57–79)	69 (56–79)	69 (55–79)	67 (52–78)
Male Sex (n, %)	64010 (57.9%)	5267 (59.2%)	3785 (60.1%)	14244 (58.2%)	17444 (58.0%)	13095 (57.2%)	10175 (56.4%)
SAPS3 (median, IQR)	51 (41–63)	51 (40–63)	52 (41–65)	51.0 (42–63)	51 (42–63)	51 (41–63)	51 (41–63)
Admission type (n, %)							
medical	80602 (72.9%)	6108 (68.7%)	5053 (80.3%)	19931 (81.5%)	22166 (73.7%)	15277 (66.8%)	12067 (66.9%)
non-scheduled surgery	30026 (27.1%)	2789 (31.3%)	1241 (19.7%)	4531 (18.5%)	7900 (26.3%)	7605 (33.2%)	5960 (33.1%)
ICU length of stay [hours] (median, IQR)							
all patients	49 (21–142)	41 (12–132)	40 (12–124)	50 (24–144)	51 (22–148)	51 (19–141)	42 (15–133)
ICU survivors	49 (21–136)	38 (12–129)	41 (14–123)	50 (24–127)	49 (22–143)	48 (19–138)	41 (15–115)
ICU non-survivors	61 (15–204)	58 (13–190)	37 (8–147)	58 (14–199)	74 (20–230)	61 (16–210)	61 (15–208)
ICU mortality (n, %)	14688 (13.3%)	1196 (13.4%)	1020 (16.2%)	3346 (13.7%)	3838 (12.8%)	3004 (13.1%)	2284 (12.7%)
Hospital mortality (n, %)	21009 (19.2%)	1644 (18.7%)	1347 (21.7%)	4768 (19.7%)	5635 (18.9%)	4324 (19.1%)	3291 (18.4%)

### Descriptive statistics

**Figure 2:**
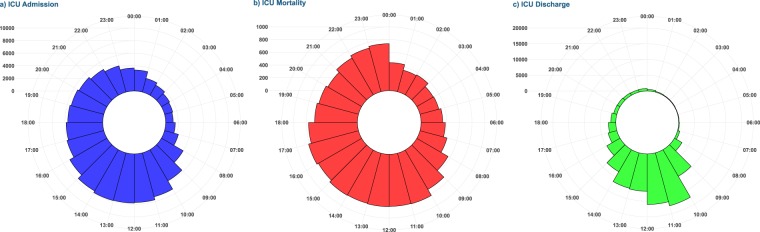
Timing of (**a**) ICU admissions, (**b**) ICU discharges and (**c**) death in the ICU.

Event rates of admission, discharge and death were presented in Fig. 2. Most patients were admitted to ICU during usual work hours: 54,528 (49.3%) were admitted between 08:00 and 15:59. Late night and early morning hours were noticeably less busy; only 15,191 (13.7%) patients were admitted to ICU between 00:00 and 07:59. General demographics and severity of illness varied little between patients admitted during the course of the day. A higher rate of patients referred to intensive care after urgent or emergent surgical procedures was observed in the afternoon and during the night compared to regular work hours.

The highest unadjusted rate of death in the ICU was found in patients admitted between 04:00 and 07:59 (16.2%) representing an absolute risk difference of 2.5% compared to the morning reference group. Conversely, the rate of death while present in the ICU was lowest during late night and highest in the morning.

Noticeable differences in ICU length of stay between patients admitted during the night and in the early morning compared to those admitted during regular work hours and in the afternoon were detected in both ICU survivors and non-survivors: patients admitted during the day remained in the ICU longer than those admitted at night. The vast majority of patient discharges from the ICU were conducted during the morning and around midday, while only a fraction of all patients was discharged in the afternoon or during the night.

### Main analysis

**Figure 3:**
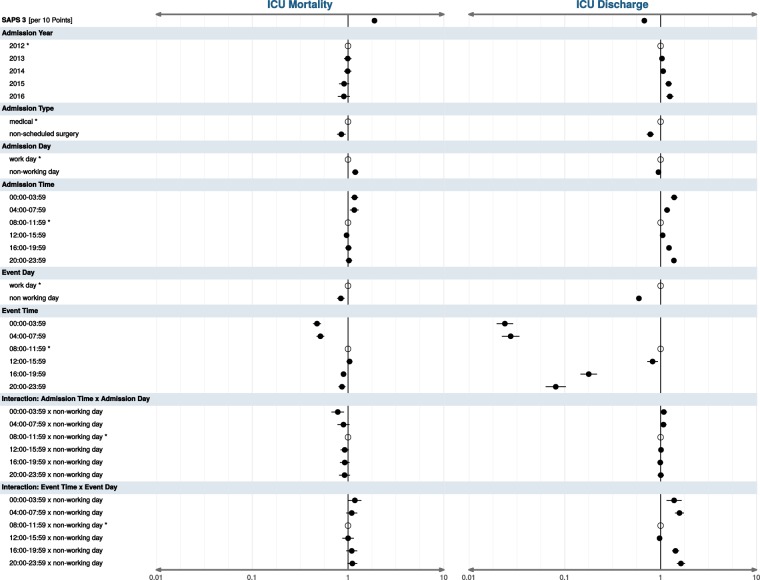
Main analysis (Fine-and-Gray-model), adjusted subdistribution HR, 95% CI for ICU mortality and ICU discharge within 30 days (n = 110,628); CI = confidence interval, HR = hazard ratio, ICU = intensive care unit, SAPS = Simplified Acute Physiology Score; covariate “admission month” not depicted.

In our multivariable competing risk analysis model for the outcomes “death in the ICU” and “discharge from the ICU” within 30 days, statistically significant “time effects” were found (Fig. 3, full model estimates and confidence intervals in Table S1). These effects concerned both the time of admission and the time of the outcomes under investigation. Predictive power of the chosen explanatory variables was adequate (C-statistics = 0.816 for ICU mortality; 0.820 for ICU discharge).

Admission to intensive care units during late night and early morning (00:00–07:59) was associated with increased hazards of death in the ICU; these were HR: 1.17; 95% CI: 1.08–1.28 for the 00:00–03:59 interval and HR: 1.16; 95% CI: 1.05–1.29 for 04:00–07:59. Mortality risk was practically identical for admissions between 08:00 and 23:59. At the same time, admission time was also associated with significant variation in the hazards for discharge from the ICU, which were lowest in the reference interval and highest in the 20:00–23:59 and 00:00–03:59 intervals.

Conversely, hazards of both events of interest occurring in the evening or at nights were drastically lower than during the day. Risk of death in the ICU was stable between 08:00 and 15:59 but decreased steadily over the rest of the day; lowest HR for death in the ICU were found between 00:00 and 07:59. Similarly, hazards for discharge from the ICU were highest in the reference time period (08:00–11:59) but dropped sharply over the course of the day.

The occurrence of previously described “weekend effects” was reaffirmed. Admission to intensive care at non-working days was associated with increased hazards of death in the ICU, HR: 1.19; 95% CI: 1.10–1.29. Concurrently, event rates at non-working days were lower than at work days; these were HR: 0.84, 95% CI: 0.77–0.92 for death in the ICU and HR: 0.59; 95% CI: 0.56–0.63 for discharge from the ICU.

The interaction terms between admission time and admission day type demonstrated a slight attenuation of the aforementioned associations between admission time and hazards of death during late night at non-working days. There was a noticeable interaction between time of discharge and day type: discharge from the ICU in the late afternoon or at night was more likely at non-working days than at work days.

### Sensitivity analyses

All time-associated effects described earlier could also be demonstrated in sensitivity analyses in cohorts including readmitted patients (Table S2) and in all patients including those admitted to ICU after scheduled and unspecified surgical procedures (Table S3). Additionally, there were significant interactions between the time and the day type of admission in this model: increased hazards of death following evening and night time admission to the ICU were attenuated on non-working days. Only minute numerical differences to the main model’s results were found in sensitivity analyses using different definitions of non-working days and time beginnings and endings (Tables S4–S6).

According to Schoenfeld’s residuals the model adhered closer to the proportional hazards assumption for ICU mortality than for ICU discharge. The addition of an interaction term between time of day of admission and time of day of event to the model for ICU discharge improved the residual pattern slightly. Effects found in the main model were pronounced (Table S7), yet concordance remained almost identical (C-statistics = 0.821). The more parsimonious model was presented as the main model. All described effects could be reproduced in the Cox-model (Table S8).

## Discussion

This study confirms the assumption that there are “time effects” in intensive care units. These effects seem to exist in parallel to previously described “weekend effects”. Comparisons between patients admitted at very different times during the day are prone to bias due to variation in case-mix and severity of illness. We therefore focused our analysis on urgency and emergency admissions to intensive care units, i.e. those that occur all over the day. We also adjusted for severity of illness using the well-established SAPS 3 score.

Discernible “time effects” were manifold. First and foremost, ICU admission during late night and early mornings was associated with increased hazards of death. Since our analysis was conducted in a relatively homogenous patient cohort and was adjusted for severity of illness, we do not solely attribute these findings to variation in case-mix. Concurrently, use of our competing-risk model yielded a seemingly counterintuitive finding: hazard ratios for alive discharge from the ICU were higher in patients admitted during night time than in those admitted during the day. Both findings may in part be explained by time-to-event analyses: since times to discharge from the ICU in survivors and time to death in non-survivors were significantly shorter following night time admission to intensive care, hazards are elevated in these admission time intervals.

This can be explained by another key finding of our study: far more decisions regarding patient management are made during work hours. The fact that patient transfers from intensive care units to wards and other units of further care almost exclusively occur during normal work hours during the week may be regarded as proof of that assumption. Concomitantly, the hazards for death in the ICU were lower in the late afternoon and early night hours and drastically lower during late night and early morning when compared to the regular work hours. While there may indeed be regulatory processes in human physiology following a circadian rhythm that may explain these findings, it seems more plausible that these effects are due to the need of senior decision making ahead of possible demise of critically ill patients. These decisions are more likely to be made during regular working hours, when senior staff from all health care professions are physically present.

The requirement for, and consequences of, these active decisions leads to variation in time elapsed between admission to intensive care and the occurrence of the final event, be it discharge from the ICU or death in the ICU. The observed changes of hazards of death and discharge in a time-to-event analysis may therefore be system-inherent and system-driven rather than due to inadequacy of care provided at the time of admission.

Furthermore, an effect already demonstrated previously was reaffirmed: the probability of discharge from ICU is drastically lower at weekends than during the week. However, a time-associated facet was detected: the decrease in the rate of ICU discharge in the afternoon and night is attenuated at weekends, represented by the interaction between event time and event day in the main analysis. This may be due to an increase in unplanned ICU discharges during these hours that becomes necessary due to low chances of discharge at weekends and concomitant scarcity of intensive care capacity.

Findings from this study are in line with some previous studies, while they conflict with others. Most notably, studies conducted in large data sets in Australia and New Zealand^13^, Canada^4^, the Netherlands^12^, Poland^6^, the United Kingdom^8^ and the United States^11^ reported increased risk of death in patients admitted to ICU “off hours”. On the contrary, a study in a large cohort from the United Kingdom^14^ and other, mostly smaller, studies^2,3,5,7,9^ did not detect associations of ICU admission time and outcomes. The comparison of these studies is hampered by the various methodologic approaches used, especially with regards to adjustment for severity of illness, and due to inconsistent definitions of “on hours” and “off hours”.

A recent systematic review and meta-analysis has sought to circumvent most of these issues and accumulate the existing body of knowledge^15^. Analysing 717,331 patients from 14 studies, the authors conclude that there was no overall association between “off hours” admission to ICU and outcomes, although they found significant heterogeneity between studies. The present study adds findings of higher granularity and generalisability, as it divides time of day into four-hour blocks instead of using arbitrary divisions. Furthermore, our approach and findings go beyond previous efforts to identify “time effects” solely focusing on time of ICU admission. We therefore present broader insights into the workings of intensive care units around the clock and associated outcomes.

### Strengths

Use of a competing risk analysis approach enabled us to specifically investigate the influence exerted on patient outcomes by intensive care itself. Statistical validity is inferred by good predictive power and stability of results in both the Fine-and-Gray subdistribution hazards model and Cox cause-specific hazard models. The observed concordance for all analyses were all well within the accepted limits in heterogeneous populations such as the critically ill^25,26^. Comparably large sample size and completeness of reporting due to Austrian healthcare legislation requiring reporting of key items in all patients admitted to intensive care are further notable strengths of this study.

### Limitations

This study’s findings are based on a retrospective analysis of data queried from a prospectively gathered database using a multivariable competing risk model for time-until-event data. It is therefore subject to all limitations that apply to this study type. Documentation and coding are in the responsibility of individual health care providers and may be incomplete, especially if data input is not required by law or local policy. Data regarding treatment and outcomes after discharge from intensive care may be limited, as they are out of scope of the ASDI database. Patient heterogeneity and variation in case-mix may obviously bias findings. We therefore adjusted for baseline severity of illness using the SAPS 3 system. Possible limitations of this scoring system may apply to this study. Findings from this study are not necessarily generalizable to other health systems.

## Conclusion

Night time admission to intensive care units is associated with increased hazards of death following adjustment for severity of illness. Death in the ICU and discharge from the ICU are less likely during night time. These findings likely reflect how decision-making affects outcomes and the time of their occurrence in critically ill patients, and how physiology can be upheld by modern intensive care medicine at times, when these decision-making processes are less likely to occur.

## Declarations

### Ethics approval and consent to participate

This study was conducted according to the ethical standards laid down in the 1964 Declaration of Helsinki and its later amendments. The ethics committee at the Medical University of Graz, Austria, IRB00002556, approved of the study (30–054 ex 17/18) before its conduction. The need for informed consent was waived by the institutional review board since no additional interventions were performed.

## Supplementary information

Supplementary Material

## Data Availability

The datasets used and/or analysed during the current study are available from the corresponding author on reasonable request.
