# Supplementary material for: Time of Day and its Association with Risk of Death and Chance of Discharge in Critically Ill Patients: A Retrospective Study
Source: Sci Rep. 2019 Aug 29;9:12533. doi: 10.1038/s41598-019-48947-y (PMC6715801; doi:10.1038/s41598-019-48947-y)
Supplement: Supplementary file 1 — Supplementary Material [file 41598_2019_48947_MOESM1_ESM.pdf]

SUPPLEMENTARY MATERIAL

Time of Day and its Association with Risk of Death and Chance of Discharge in Critically Ill Patients: A Retrospective Study

Paul Zajic[1]
Peter Bauer[2]
Andrew Rhodes[3]
Rui Moreno[4]
Tobias Fellinger[2]
Barbara Metnitz[5]
Martin Posch[2]
Philipp G. H. Metnitz[1]

[1] Div. of General Anaesthesiology, Emergency- and Intensive Care Medicine Medical University of Graz, Graz, Austria

[2] Centre for Medical Statistics, Informatics, and Intelligent Systems, Medical University of Vienna, Vienna, Austria

[3] St George's University Hospitals NHS Foundation Trust, St George's University of London, London, United Kingdom

[4] Unidade de Cuidados Intensivos Neurocríticos, Centro Hospitalar de Lisboa Central, Lisbon, Portugal

[5] Austrian Centre for Documentation and Quality Assurance in Intensive Care, Vienna, Austria

TABLE OF CONTENTS

Main Analysis: Fine-and-Gray Model 1

Sensitivity Analysis: Main Cohort including Readmissions 2

Sensitivity Analysis: Extended Cohort 3

Sensitivity Analysis: Alternative Weekday Beginnings and Endings 4

Sensitivity Analysis: Alternative Non-Working Day Definitions 5

Sensitivity Analysis: Alternate Daytime and Non-Working Day Definitions 6

Sensitivity Analysis: Fine-and-Gray Model with additional Interaction 7

Sensitivity Analysis: Main Cohort analysed using Cox-Model 9

MAIN ANALYSIS: FINE-AND-GRAY MODEL

Table S 1 Main Analysis using the Fine-and-Gray model described in the manuscript; admission month not shown

	ICU MORTALITY			ICU DISCHARGE		
	**HR**	**95% CI**	***p***	**HR**	**95% CI**	***p***
**SAPS3** [per 10 points]	1.889	(1.826 - 1.955)	<0.001	0.676	(0.659 - 0.694)	<0.001
**Admission Year**						
2012 *	1.000			1.000		
2013	0.993	(0.909 - 1.085)	0.883	1.034	(0.970 - 1.101)	0.303
2014	0.992	(0.908 - 1.083)	0.852	1.062	(0.989 - 1.140)	0.096
2015	0.909	(0.805 - 1.027)	0.126	1.212	(1.118 - 1.315)	<0.001
2016	0.905	(0.784 - 1.045)	0.174	1.249	(1.147 - 1.361)	<0.001
**Admission Type**						
medical *	1.000			1.000		
non-scheduled surgery	0.852	(0.768 - 0.945)	0.002	0.781	(0.716 - 0.853)	<0.001
**Admission Day**						
work day *	1.000			1.000		
non-working day	1.191	(1.103 - 1.286)	<0.001	0.950	(0.892 - 1.011)	0.109
**Admission Time**						
00:00 – 03:59	1.172	(1.076 - 1.275)	<0.001	1.386	(1.282 - 1.497)	<0.001
04:00 – 07:59	1.163	(1.046 - 1.292)	0.005	1.168	(1.087 - 1.256)	<0.001
08:00 – 11:59 *	1.000			1.000		
12:00 – 15:59	0.966	(0.903 - 1.033)	0.313	1.050	(1.009 - 1.093)	0.016
16:00 – 19:59	1.011	(0.936 - 1.092)	0.779	1.224	(1.158 - 1.293)	<0.001
20:00 – 23:59	1.023	(0.947 - 1.106)	0.565	1.377	(1.291 - 1.468)	<0.001
**Event Day**						
work day *	1.000			1.000		
non-working day	0.842	(0.770 - 0.920)	<0.001	0.594	(0.561 - 0.629)	<0.001
**Event Time**						
00:00 – 03:59	0.475	(0.432 - 0.522)	<0.001	0.024	(0.019 - 0.029)	<0.001
04:00 – 07:59	0.515	(0.470 - 0.565)	<0.001	0.027	(0.022 - 0.034)	<0.001
08:00 – 11:59 *	1.000			1.000		
12:00 – 15:59	1.038	(0.965 - 1.116)	0.315	0.825	(0.726 - 0.938)	0.003
16:00 – 19:59	0.898	(0.839 - 0.960)	0.002	0.178	(0.146 - 0.217)	<0.001
20:00 – 23:59	0.866	(0.794 - 0.943)	0.001	0.080	(0.063 - 0.103)	<0.001
**Interaction: Admission Time x Admission Day**						
00:00 – 03:59 x non-working day	0.782	(0.672 - 0.910)	0.001	1.079	(0.998 - 1.168)	0.057
04:00 – 07:59 x non-working day	0.897	(0.776 - 1.037)	0.142	1.071	(0.994 - 1.153)	0.070
08:00 – 11:59 x non-working day *	1.000			1.000		
12:00 – 15:59 x non-working day	0.923	(0.835 - 1.021)	0.121	1.010	(0.949 - 1.076)	0.752
16:00 – 19:59 x non-working day	0.924	(0.824 - 1.038)	0.183	0.992	(0.926 - 1.063)	0.818
20:00 – 23:59 x non-working day	0.920	(0.807 - 1.050)	0.215	1.004	(0.930 - 1.085)	0.910
**Interaction: Event Time x Event Day**						
00:00 – 03:59 x non-working day	1.179	(1.010 - 1.376)	0.037	1.384	(1.154 - 1.659)	<0.001
04:00 – 07:59 x non-working day	1.093	(0.958 - 1.248)	0.185	1.575	(1.416 - 1.751)	<0.001
08:00 – 11:59 x non-working day *	1.000			1.000		
12:00 – 15:59 x non-working day	1.002	(0.873 - 1.149)	0.978	0.975	(0.918 - 1.037)	0.424
16:00 – 19:59 x non-working day	1.094	(0.957 - 1.250)	0.187	1.431	(1.316 - 1.557)	<0.001
20:00 – 23:59 x non-working day	1.111	(0.987 - 1.249)	0.081	1.630	(1.478 - 1.799)	<0.001

SENSITIVITY ANALYSIS: MAIN COHORT INCLUDING READMISSIONS

Table S 2 Analysis using the Fine-and-Gray model on the main cohort, including all patients readmitted to the ICU

	ICU MORTALITY			ICU DISCHARGE		
	**HR**	**95% CI**	***p***	**HR**	**95% CI**	***p***
**SAPS3** [per 10 points]	1.875	(1.811 - 1.942)	<0.001	0.681	(0.664 - 0.698)	<0.001
**Admission Year**						
2012 *	1.000			1.000		
2013	0.997	(0.912 - 1.090)	0.945	1.036	(0.973 - 1.104)	0.267
2014	0.975	(0.896 - 1.062)	0.562	1.066	(0.994 - 1.143)	0.072
2015	0.898	(0.791 - 1.019)	0.095	1.224	(1.126 - 1.331)	<0.001
2016	0.895	(0.775 - 1.033)	0.128	1.258	(1.153 - 1.373)	<0.001
**Admission Type**						
medical *	1.000			1.000		
non-scheduled surgery	0.863	(0.779 - 0.957)	0.005	0.787	(0.723 - 0.858)	<0.001
**Admission Day**						
work day *	1.000			1.000		
non-working day	1.158	(1.075 - 1.247)	<0.001	0.960	(0.908 - 1.016)	0.156
**Admission Time**						
00:00 – 03:59	1.168	(1.078 - 1.266)	<0.001	1.399	(1.299 - 1.507)	<0.001
04:00 – 07:59	1.162	(1.054 - 1.281)	0.003	1.193	(1.115 - 1.275)	<0.001
08:00 – 11:59 *	1.000			1.000		
12:00 – 15:59	0.977	(0.920 - 1.037)	0.440	1.043	(1.004 - 1.085)	0.032
16:00 – 19:59	1.020	(0.951 - 1.095)	0.577	1.237	(1.171 - 1.308)	<0.001
20:00 – 23:59	1.014	(0.944 - 1.090)	0.704	1.397	(1.311 - 1.489)	<0.001
**Event Day**						
work day *	1.000			1.000		
non-working day	0.850	(0.782 - 0.923)	<0.001	0.578	(0.545 - 0.612)	<0.001
**Event Time**						
00:00 – 03:59	0.482	(0.439 - 0.528)	<0.001	0.023	(0.019 - 0.027)	<0.001
04:00 – 07:59	0.511	(0.468 - 0.558)	<0.001	0.027	(0.022 - 0.034)	<0.001
08:00 – 11:59 *	1.000			1.000		
12:00 – 15:59	1.027	(0.960 - 1.099)	0.445	0.816	(0.721 - 0.924)	0.001
16:00 – 19:59	0.883	(0.828 - 0.941)	<0.001	0.173	(0.142 - 0.210)	<0.001
20:00 – 23:59	0.850	(0.783 - 0.924)	<0.001	0.078	(0.062 - 0.099)	<0.001
**Interaction: Admission Time x Admission Day**						
00:00 – 03:59 x non-working day	0.835	(0.723 - 0.965)	0.014	1.064	(0.993 - 1.141)	0.080
04:00 – 07:59 x non-working day	0.885	(0.768 - 1.020)	0.093	1.048	(0.978 - 1.123)	0.184
08:00 – 11:59 x non-working day *	1.000			1.000		
12:00 – 15:59 x non-working day	0.947	(0.859 - 1.043)	0.269	1.010	(0.955 - 1.069)	0.717
16:00 – 19:59 x non-working day	0.942	(0.843 - 1.053)	0.291	0.979	(0.921 - 1.041)	0.501
20:00 – 23:59 x non-working day	0.949	(0.835 - 1.078)	0.422	0.985	(0.917 - 1.058)	0.675
**Interaction: Event Time x Event Day**						
00:00 – 03:59 x non-working day	1.164	(1.013 - 1.337)	0.032	1.478	(1.258 - 1.736)	<0.001
04:00 – 07:59 x non-working day	1.133	(1.000 - 1.283)	0.051	1.593	(1.424 - 1.781)	<0.001
08:00 – 11:59 x non-working day *	1.000			1.000		
12:00 – 15:59 x non-working day	0.989	(0.867 - 1.128)	0.865	0.988	(0.928 - 1.052)	0.711
16:00 – 19:59 x non-working day	1.107	(0.976 - 1.256)	0.113	1.466	(1.353 - 1.587)	<0.001
20:00 – 23:59 x non-working day	1.105	(0.995 - 1.228)	0.062	1.677	(1.520 - 1.849)	<0.001

SENSITIVITY ANALYSIS: EXTENDED COHORT

Table S 3 Analysis using the Fine-and-Gray model on all adult patients (i.e. including scheduled admissions)

	ICU MORTALITY			ICU DISCHARGE		
	**HR**	**95% CI**	***p***	**HR**	**95% CI**	***p***
**SAPS3** [per 10 points]	1.942	(1.875 - 2.011)	<0.001	0.692	(0.673 - 0.711)	<0.001
**Admission Year**						
2012 *	1.000			1.000		
2013	0.970	(0.900 - 1.046)	0.432	1.046	(1.001 - 1.094)	0.047
2014	0.975	(0.898 - 1.059)	0.545	1.072	(1.012 - 1.136)	0.018
2015	0.904	(0.804 - 1.016)	0.090	1.147	(1.078 - 1.220)	<0.001
2016	0.903	(0.785 - 1.039)	0.153	1.179	(1.101 - 1.263)	<0.001
**Admission Type**						
medical *	1.000			1.000		
non-scheduled surgery	0.855	(0.770 - 0.950)	0.003	0.786	(0.720 - 0.859)	<0.001
scheduled surgery	0.510	(0.419 - 0.619)	<0.001	0.985	(0.869 - 1.116)	0.814
unspecified surgery	1.282	(1.019 - 1.612)	0.034	0.986	(0.851 - 1.142)	0.847
**Admission Day**						
work day *	1.000			1.000		
non-working day	1.267	(1.174 - 1.367)	<0.001	0.874	(0.813 - 0.940)	<0.001
**Admission Time**						
00:00 – 03:59	1.271	(1.169 - 1.381)	<0.001	1.588	(1.441 - 1.750)	<0.001
04:00 – 07:59	1.215	(1.097 - 1.346)	<0.001	1.156	(1.083 - 1.235)	<0.001
08:00 – 11:59 *	1.000			1.000		
12:00 – 15:59	0.996	(0.933 - 1.063)	0.893	1.095	(1.029 - 1.166)	0.004
16:00 – 19:59	1.088	(1.010 - 1.172)	0.026	1.270	(1.166 - 1.383)	<0.001
20:00 – 23:59	1.122	(1.037 - 1.215)	0.004	1.516	(1.382 - 1.664)	<0.001
**Event Day**						
work day *	1.000			1.000		
non-working day	0.841	(0.775 - 0.912)	<0.001	0.567	(0.527 - 0.609)	<0.001
**Event Time**						
00:00 – 03:59	0.483	(0.442 - 0.529)	<0.001	0.017	(0.014 - 0.021)	<0.001
04:00 – 07:59	0.528	(0.483 - 0.577)	<0.001	0.021	(0.017 - 0.027)	<0.001
08:00 – 11:59 *	1.000			1.000		
12:00 – 15:59	1.024	(0.956 - 1.097)	0.492	0.604	(0.523 - 0.698)	<0.001
16:00 – 19:59	0.896	(0.840 - 0.956)	0.001	0.121	(0.100 - 0.148)	<0.001
20:00 – 23:59	0.876	(0.808 - 0.950)	0.001	0.057	(0.046 - 0.071)	<0.001
**Interaction: Admission Time x Admission Day**						
00:00 – 03:59 x non-working day	0.733	(0.639 - 0.840)	<0.001	1.182	(1.091 - 1.281)	<0.001
04:00 – 07:59 x non-working day	0.864	(0.744 - 1.002)	0.053	1.154	(1.069 - 1.247)	<0.001
08:00 – 11:59 x non-working day *	1.000			1.000		
12:00 – 15:59 x non-working day	0.914	(0.827 - 1.011)	0.080	0.993	(0.929 - 1.061)	0.830
16:00 – 19:59 x non-working day	0.862	(0.770 - 0.964)	0.010	1.046	(0.964 - 1.136)	0.282
20:00 – 23:59 x non-working day	0.845	(0.741 - 0.963)	0.012	1.108	(1.018 - 1.205)	0.018
**Interaction: Event Time x Event Day**						
00:00 – 03:59 x non-working day	1.184	(1.029 - 1.362)	0.018	1.701	(1.449 - 1.996)	<0.001
04:00 – 07:59 x non-working day	1.098	(0.973 - 1.239)	0.131	1.693	(1.460 - 1.964)	<0.001
08:00 – 11:59 x non-working day *	1.000			1.000		
12:00 – 15:59 x non-working day	1.047	(0.922 - 1.188)	0.479	1.123	(1.046 - 1.206)	0.001
16:00 – 19:59 x non-working day	1.098	(0.961 - 1.254)	0.167	1.583	(1.452 - 1.725)	<0.001
20:00 – 23:59 x non-working day	1.090	(0.983 - 1.209)	0.100	1.861	(1.671 - 2.074)	<0.001

SENSITIVITY ANALYSIS: ALTERNATIVE WEEKDAY BEGINNINGS AND ENDINGS

Table S 4 Analysis using the Fine-and-Gray model with alternate weekday definitions (i.e. 00:00 – 23:59 instead of 08:00 – 07:59)

	ICU MORTALITY			ICU DISCHARGE		
	**HR**	**95% CI**	***p***	**HR**	**95% CI**	***p***
**SAPS3** [per 10 points]	1.889	(1.825 - 1.955)	<0.001	0.676	(0.660 - 0.694)	<0.001
**Admission Year**						
2012 *	1.000			1.000		
2013	0.994	(0.910 - 1.086)	0.889	1.034	(0.971 - 1.101)	0.299
2014	0.992	(0.908 - 1.083)	0.850	1.062	(0.990 - 1.140)	0.094
2015	0.909	(0.805 - 1.027)	0.127	1.213	(1.118 - 1.315)	<0.001
2016	0.904	(0.783 - 1.044)	0.170	1.250	(1.147 - 1.361)	<0.001
**Admission Type**						
medical *	1.000			1.000		
non-scheduled surgery	0.852	(0.768 - 0.946)	0.003	0.781	(0.716 - 0.852)	<0.001
**Admission Day**						
work day *	1.000			1.000		
non-working day	1.190	(1.101 - 1.285)	<0.001	0.950	(0.892 - 1.011)	0.108
**Admission Time**						
00:00 – 03:59	1.138	(1.049 - 1.235)	0.002	1.357	(1.258 - 1.463)	<0.001
04:00 – 07:59	1.138	(1.026 - 1.264)	0.015	1.150	(1.071 - 1.235)	<0.001
08:00 – 11:59 *	1.000			1.000		
12:00 – 15:59	0.966	(0.903 - 1.033)	0.312	1.050	(1.009 - 1.093)	0.016
16:00 – 19:59	1.011	(0.936 - 1.092)	0.778	1.224	(1.159 - 1.293)	<0.001
20:00 – 23:59	1.023	(0.947 - 1.106)	0.561	1.377	(1.292 - 1.469)	<0.001
**Event Day**						
work day *	1.000			1.000		
non-working day	0.838	(0.766 - 0.916)	<0.001	0.592	(0.559 - 0.626)	<0.001
**Event Time**						
00:00 – 03:59	0.475	(0.434 - 0.521)	<0.001	0.023	(0.019 - 0.027)	<0.001
04:00 – 07:59	0.519	(0.475 - 0.566)	<0.001	0.028	(0.022 - 0.035)	<0.001
08:00 – 11:59 *	1.000			1.000		
12:00 – 15:59	1.037	(0.965 - 1.115)	0.323	0.825	(0.726 - 0.938)	0.003
16:00 – 19:59	0.897	(0.838 - 0.960)	0.002	0.178	(0.146 - 0.217)	<0.001
20:00 – 23:59	0.865	(0.793 - 0.943)	0.001	0.080	(0.063 - 0.103)	<0.001
**Interaction: Admission Time x Admission Day**						
00:00 – 03:59 x non-working day	0.860	(0.757 - 0.976)	0.019	1.150	(1.061 - 1.247)	0.001
04:00 – 07:59 x non-working day	0.966	(0.817 - 1.143)	0.690	1.131	(1.046 - 1.222)	0.002
08:00 – 11:59 x non-working day *	1.000			1.000		
12:00 – 15:59 x non-working day	0.923	(0.835 - 1.021)	0.120	1.010	(0.948 - 1.075)	0.762
16:00 – 19:59 x non-working day	0.924	(0.823 - 1.037)	0.179	0.991	(0.925 - 1.062)	0.801
20:00 – 23:59 x non-working day	0.919	(0.806 - 1.049)	0.211	1.004	(0.929 - 1.084)	0.928
**Interaction: Event Time x Event Day**						
00:00 – 03:59 x non-working day	1.175	(1.038 - 1.330)	0.011	1.638	(1.394 - 1.923)	<0.001
04:00 – 07:59 x non-working day	1.068	(0.935 - 1.221)	0.331	1.502	(1.269 - 1.777)	<0.001
08:00 – 11:59 x non-working day *	1.000			1.000		
12:00 – 15:59 x non-working day	1.004	(0.875 - 1.152)	0.953	0.976	(0.919 - 1.037)	0.435
16:00 – 19:59 x non-working day	1.097	(0.960 - 1.253)	0.174	1.433	(1.318 - 1.558)	<0.001
20:00 – 23:59 x non-working day	1.114	(0.990 - 1.254)	0.073	1.632	(1.479 - 1.801)	<0.001

SENSITIVITY ANALYSIS: ALTERNATIVE NON-WORKING DAY DEFINITIONS

Table S 5 Analysis using the Fine-and-Gray model with weekends (Saturday and Sunday) as non-working days only

	ICU MORTALITY			ICU DISCHARGE		
	**HR**	**95% CI**	***p***	**HR**	**95% CI**	***p***
**SAPS3** [per 10 points]	1.889	(1.825 - 1.955)	<0.001	0.676	(0.660 - 0.694)	<0.001
**Admission Year**						
2012 *	1.000			1.000		
2013	0.993	(0.909 - 1.085)	0.878	1.035	(0.972 - 1.103)	0.282
2014	0.991	(0.908 - 1.082)	0.842	1.065	(0.992 - 1.142)	0.082
2015	0.909	(0.805 - 1.027)	0.124	1.214	(1.119 - 1.317)	<0.001
2016	0.904	(0.783 - 1.045)	0.171	1.253	(1.150 - 1.364)	<0.001
**Admission Type**						
medical *	1.000			1.000		
non-scheduled surgery	0.852	(0.768 - 0.945)	0.002	0.782	(0.716 - 0.853)	<0.001
**Admission Day**						
work day *	1.000			1.000		
non-working day	1.193	(1.102 - 1.293)	<0.001	0.954	(0.894 - 1.019)	0.162
**Admission Time**						
00:00 – 03:59	1.100	(1.004 - 1.204)	0.041	1.390	(1.286 - 1.502)	<0.001
04:00 – 07:59	1.177	(1.057 - 1.311)	0.003	1.155	(1.072 - 1.245)	<0.001
08:00 – 11:59 *	1.000			1.000		
12:00 – 15:59	0.966	(0.904 - 1.032)	0.300	1.051	(1.011 - 1.094)	0.013
16:00 – 19:59	1.017	(0.945 - 1.094)	0.658	1.224	(1.160 - 1.292)	<0.001
20:00 – 23:59	1.030	(0.955 - 1.111)	0.437	1.374	(1.289 - 1.464)	<0.001
**Event Day**						
work day *	1.000			1.000		
non-working day	0.839	(0.770 - 0.915)	<0.001	0.593	(0.560 - 0.628)	<0.001
**Event Time**						
00:00 – 03:59	0.469	(0.431 - 0.511)	<0.001	0.022	(0.018 - 0.027)	<0.001
04:00 – 07:59	0.493	(0.457 - 0.531)	<0.001	0.028	(0.022 - 0.035)	<0.001
08:00 – 11:59 *	1.000			1.000		
12:00 – 15:59	1.041	(0.968 - 1.119)	0.279	0.826	(0.727 - 0.939)	0.004
16:00 – 19:59	0.900	(0.840 - 0.965)	0.003	0.180	(0.147 - 0.220)	<0.001
20:00 – 23:59	0.866	(0.795 - 0.943)	0.001	0.081	(0.064 - 0.104)	<0.001
**Interaction: Admission Time x Admission Day**						
00:00 – 03:59 x non-working day	0.955	(0.812 - 1.123)	0.579	1.069	(0.965 - 1.183)	0.200
04:00 – 07:59 x non-working day	0.852	(0.717 - 1.013)	0.069	1.119	(1.018 - 1.230)	0.020
08:00 – 11:59 x non-working day *	1.000			1.000		
12:00 – 15:59 x non-working day	0.920	(0.832 - 1.016)	0.099	1.005	(0.942 - 1.072)	0.882
16:00 – 19:59 x non-working day	0.901	(0.804 - 1.010)	0.074	0.987	(0.920 - 1.059)	0.716
20:00 – 23:59 x non-working day	0.894	(0.786 - 1.017)	0.088	1.010	(0.933 - 1.093)	0.807
**Interaction: Event Time x Event Day**						
00:00 – 03:59 x non-working day	1.241	(1.100 - 1.400)	<0.001	1.795	(1.522 - 2.118)	<0.001
04:00 – 07:59 x non-working day	1.285	(1.125 - 1.468)	<0.001	1.469	(1.253 - 1.722)	<0.001
08:00 – 11:59 x non-working day *	1.000			1.000		
12:00 – 15:59 x non-working day	0.991	(0.870 - 1.130)	0.894	0.966	(0.906 - 1.029)	0.283
16:00 – 19:59 x non-working day	1.087	(0.945 - 1.250)	0.243	1.408	(1.285 - 1.542)	<0.001
20:00 – 23:59 x non-working day	1.118	(1.000 - 1.250)	0.050	1.608	(1.448 - 1.787)	<0.001

SENSITIVITY ANALYSIS: ALTERNATE DAYTIME AND NON-WORKING DAY DEFINITIONS

Table S 6 Analysis using the Fine-and-Gray model with weekends as non-working days and 00:00 – 23:59 as weekday cutoffs

	ICU MORTALITY			ICU DISCHARGE		
	**HR**	**95% CI**	***p***	**HR**	**95% CI**	***p***
**SAPS3** [per 10 points]	1.889	(1.825 - 1.955)	<0.001	0.677	(0.660 - 0.694)	<0.001
**Admission Year**						
2012 *	1.000			1.000		
2013	0.994	(0.910 - 1.086)	0.891	1.035	(0.972 - 1.103)	0.279
2014	0.992	(0.908 - 1.083)	0.850	1.065	(0.992 - 1.143)	0.081
2015	0.910	(0.805 - 1.027)	0.127	1.215	(1.120 - 1.317)	<0.001
2016	0.904	(0.783 - 1.045)	0.172	1.253	(1.150 - 1.364)	<0.001
**Admission Type**						
medical *	1.000			1.000		
non-scheduled surgery	0.852	(0.768 - 0.945)	0.002	0.782	(0.716 - 0.853)	<0.001
**Admission Day**						
work day *	1.000			1.000		
non-working day	1.191	(1.099 - 1.291)	<0.001	0.955	(0.894 - 1.020)	0.168
**Admission Time**						
00:00 – 03:59	1.147	(1.058 - 1.244)	0.001	1.359	(1.260 - 1.465)	<0.001
04:00 – 07:59	1.133	(1.023 - 1.256)	0.017	1.156	(1.078 - 1.241)	<0.001
08:00 – 11:59 *	1.000			1.000		
12:00 – 15:59	0.966	(0.904 - 1.031)	0.298	1.052	(1.011 - 1.094)	0.012
16:00 – 19:59	1.017	(0.944 - 1.094)	0.661	1.225	(1.160 - 1.292)	<0.001
20:00 – 23:59	1.030	(0.955 - 1.111)	0.438	1.374	(1.289 - 1.464)	<0.001
**Event Day**						
work day *	1.000			1.000		
non-working day	0.837	(0.769 - 0.912)	<0.001	0.592	(0.560 - 0.625)	<0.001
**Event Time**						
00:00 – 03:59	0.477	(0.437 - 0.522)	<0.001	0.023	(0.019 - 0.028)	<0.001
04:00 – 07:59	0.518	(0.476 - 0.564)	<0.001	0.028	(0.022 - 0.035)	<0.001
08:00 – 11:59 *	1.000			1.000		
12:00 – 15:59	1.040	(0.968 - 1.118)	0.286	0.826	(0.727 - 0.939)	0.004
16:00 – 19:59	0.900	(0.840 - 0.964)	0.003	0.180	(0.147 - 0.220)	<0.001
20:00 – 23:59	0.865	(0.794 - 0.942)	0.001	0.081	(0.064 - 0.104)	<0.001
**Interaction: Admission Time x Admission Day**						
00:00 – 03:59 x non-working day	0.831	(0.726 - 0.951)	0.007	1.151	(1.057 - 1.254)	0.001
04:00 – 07:59 x non-working day	0.981	(0.827 - 1.164)	0.827	1.117	(1.030 - 1.210)	0.007
08:00 – 11:59 x non-working day *	1.000			1.000		
12:00 – 15:59 x non-working day	0.920	(0.832 - 1.016)	0.099	1.005	(0.942 - 1.072)	0.888
16:00 – 19:59 x non-working day	0.901	(0.804 - 1.010)	0.073	0.987	(0.919 - 1.059)	0.710
20:00 – 23:59 x non-working day	0.894	(0.785 - 1.017)	0.088	1.010	(0.933 - 1.093)	0.811
**Interaction: Event Time x Event Day**						
00:00 – 03:59 x non-working day	1.171	(1.029 - 1.333)	0.017	1.682	(1.408 - 2.010)	<0.001
04:00 – 07:59 x non-working day	1.079	(0.941 - 1.237)	0.277	1.452	(1.218 - 1.731)	<0.001
08:00 – 11:59 x non-working day *	1.000			1.000		
12:00 – 15:59 x non-working day	0.993	(0.872 - 1.132)	0.920	0.966	(0.906 - 1.029)	0.283
16:00 – 19:59 x non-working day	1.089	(0.948 - 1.252)	0.229	1.409	(1.286 - 1.542)	<0.001
20:00 – 23:59 x non-working day	1.121	(1.003 - 1.254)	0.045	1.609	(1.449 - 1.787)	<0.001

SENSITIVITY ANALYSIS: FINE-AND-GRAY MODEL WITH ADDITIONAL INTERACTION

Table S 7 Analysis using the main Fine-and-Gray model with an additional interaction “event time x admission time”

	ICU MORTALITY			ICU DISCHARGE		
	**HR**	**95% CI**	***p***	**HR**	**95% CI**	***p***
**SAPS3** [per 10 points]	1.889	(1.826 - 1.955)	<0.001	0.676	(0.66 - 0.694)	<0.001
**Admission Year**						
2012 *	1.000			1.000		
2013	0.993	(0.909 - 1.085)	0.883	1.034	(0.970 - 1.101)	0.306
2014	0.992	(0.908 - 1.083)	0.852	1.062	(0.990 - 1.140)	0.095
2015	0.909	(0.805 - 1.027)	0.126	1.212	(1.117 - 1.315)	<0.001
2016	0.905	(0.784 - 1.045)	0.174	1.249	(1.147 - 1.361)	<0.001
**Admission Type**						
medical *	1.000			1.000		
non-scheduled surgery	0.852	(0.768 - 0.945)	0.002	0.781	(0.716 - 0.853)	<0.001
**Admission Day**						
work day *	1.000			1.000		
non-working day	1.191	(1.103 - 1.286)	<0.001	0.952	(0.894 - 1.014)	0.128
**Admission Time**						
00:00 – 03:59	1.172	(1.076 - 1.275)	<0.001	2.093	(1.843 - 2.378)	<0.001
04:00 – 07:59	1.163	(1.046 - 1.292)	0.005	1.216	(1.115 - 1.326)	<0.001
08:00 – 11:59 *	1.000			1.000		
12:00 – 15:59	0.966	(0.903 - 1.033)	0.313	1.231	(1.173 - 1.292)	<0.001
16:00 – 19:59	1.011	(0.936 - 1.092)	0.779	1.550	(1.436 - 1.673)	<0.001
20:00 – 23:59	1.023	(0.947 - 1.106)	0.565	1.977	(1.798 - 2.173)	<0.001
**Event Day**						
work day *	1.000			1.000		
non-working day	0.842	(0.770 - 0.920)	<0.001	0.593	(0.56 - 0.628)	<0.001
**Event Time**						
00:00 – 03:59	0.475	(0.432 - 0.522)	<0.001	0.012	(0.008 - 0.018)	<0.001
04:00 – 07:59	0.515	(0.470 - 0.565)	<0.001	0.024	(0.018 - 0.032)	<0.001
08:00 – 11:59 *	1.000			1.000		
12:00 – 15:59	1.038	(0.965 - 1.116)	0.315	1.140	(1.016 - 1.279)	0.026
16:00 – 19:59	0.898	(0.839 - 0.960)	0.002	0.355	(0.301 - 0.418)	<0.001
20:00 – 23:59	0.866	(0.794 - 0.943)	0.001	0.100	(0.080 - 0.127)	<0.001
**Interaction: Admission Time x Admission Day**						
00:00 – 03:59 x non-working day	0.782	(0.672 - 0.910)	0.001	1.079	(0.997 - 1.168)	0.061
04:00 – 07:59 x non-working day	0.897	(0.776 - 1.037)	0.142	1.073	(0.995 - 1.157)	0.066
08:00 – 11:59 x non-working day *	1.000			1.000		
12:00 – 15:59 x non-working day	0.923	(0.835 - 1.021)	0.121	1.005	(0.943 - 1.071)	0.875
16:00 – 19:59 x non-working day	0.924	(0.824 - 1.038)	0.183	0.990	(0.923 - 1.062)	0.776
20:00 – 23:59 x non-working day	0.920	(0.807 - 1.050)	0.215	1.003	(0.928 - 1.083)	0.949
**Interaction: Event Time x Event Day**						
00:00 – 03:59 x non-working day	1.179	(1.010 - 1.376)	0.037	1.322	(1.100 - 1.589)	0.003
04:00 – 07:59 x non-working day	1.093	(0.958 - 1.248)	0.185	1.548	(1.391 - 1.724)	<0.001
08:00 – 11:59 x non-working day *	1.000			1.000		
12:00 – 15:59 x non-working day	1.002	(0.873 - 1.149)	0.978	0.978	(0.920 - 1.039)	0.468
16:00 – 19:59 x non-working day	1.094	(0.957 - 1.250)	0.187	1.452	(1.336 - 1.578)	<0.001
20:00 – 23:59 x non-working day	1.111	(0.987 - 1.249)	0.081	1.633	(1.480 - 1.801)	<0.001

**Interaction: Event Time x Admission Time**			
Event 00:00 – 03:59 x Admission 00:00 – 03:59	4.408	(2.976 - 6.529)	<0.001
Event 04:00 – 07:59 x Admission 00:00 – 03:59	1.103	(0.822 - 1.481)	0.512
Event 12:00 – 15:59 x Admission 00:00 – 03:59	0.685	(0.597 - 0.786)	<0.001
Event 16:00 – 19:59 x Admission 00:00 – 03:59	0.303	(0.241 - 0.382)	<0.001
Event 20:00 – 23:59 x Admission 00:00 – 03:59	0.288	(0.224 - 0.370)	<0.001
Event 00:00 – 03:59 x Admission 04:00 – 07:59	1.024	(0.672 - 1.558)	0.913
Event 04:00 – 07:59 x Admission 04:00 – 07:59	3.246	(2.341 - 4.502)	<0.001
Event 12:00 – 15:59 x Admission 04:00 – 07:59	1.302	(1.164 - 1.458)	<0.001
Event 16:00 – 19:59 x Admission 04:00 – 07:59	0.755	(0.628 - 0.907)	0.003
Event 20:00 – 23:59 x Admission 04:00 – 07:59	0.814	(0.635 - 1.042)	0.102
Event 00:00 – 03:59 x Admission 12:00 – 15:59	1.298	(0.960 - 1.757)	0.090
Event 04:00 – 07:59 x Admission 12:00 – 15:59	0.921	(0.772 - 1.099)	0.359
Event 12:00 – 15:59 x Admission 12:00 – 15:59	0.625	(0.582 - 0.671)	<0.001
Event 16:00 – 19:59 x Admission 12:00 – 15:59	0.606	(0.539 - 0.681)	<0.001
Event 20:00 – 23:59 x Admission 12:00 – 15:59	1.057	(0.928 - 1.203)	0.403
Event 00:00 – 03:59 x Admission 16:00 – 19:59	1.714	(1.266 - 2.322)	<0.001
Event 04:00 – 07:59 x Admission 16:00 – 19:59	0.965	(0.770 - 1.211)	0.762
Event 12:00 – 15:59 x Admission 16:00 – 19:59	0.604	(0.541 - 0.675)	<0.001
Event 16:00 – 19:59 x Admission 16:00 – 19:59	0.420	(0.360 - 0.489)	<0.001
Event 20:00 – 23:59 x Admission 16:00 – 19:59	0.855	(0.686 - 1.066)	0.164
Event 00:00 – 03:59 x Admission 20:00 – 23:59	2.156	(1.534 - 3.030)	<0.001
Event 04:00 – 07:59 x Admission 20:00 – 23:59	1.118	(0.864 - 1.447)	0.397
Event 12:00 – 15:59 x Admission 20:00 – 23:59	0.604	(0.538 - 0.677)	<0.001
Event 16:00 – 19:59 x Admission 20:00 – 23:59	0.254	(0.211 - 0.306)	<0.001
Event 20:00 – 23:59 x Admission 20:00 – 23:59	0.770	(0.636 - 0.932)	0.007

SENSITIVITY ANALYSIS: MAIN COHORT ANALYSED USING COX-MODEL

Table S 8 Analysis using Cox-models fitted for competing risk analysis on the main cohort described in the manuscript

	ICU MORTALITY			ICU DISCHARGE		
	HR	95% CI	*p*	HR	95% CI	*p*
**SAPS3** [per 10 points]	1.599	(1.558 - 1.642)	<0.001	0.724	(0.705 - 0.744)	<0.001
**Admission Year**						
2012 *	1.000			1.000		
2013	0.986	(0.916 - 1.062)	0.716	1.035	(0.973 - 1.100)	0.278
2014	0.958	(0.887 - 1.034)	0.271	1.038	(0.975 - 1.106)	0.243
2015	0.961	(0.872 - 1.058)	0.415	1.183	(1.091 - 1.283)	<0.001
2016	1.024	(0.908 - 1.156)	0.694	1.244	(1.143 - 1.355)	<0.001
**Admission Type**						
medical *	1.000			1.000		
non-scheduled surgery	0.674	(0.618 - 0.735)	<0.001	0.695	(0.635 - 0.761)	<0.001
**Admission Day**						
work day *	1.000			1.000		
non-working day	1.183	(1.103 - 1.268)	<0.001	0.985	(0.923 - 1.051)	0.647
**Admission Time**						
00:00 – 03:59	1.235	(1.136 - 1.343)	<0.001	1.434	(1.324 - 1.554)	<0.001
04:00 – 07:59	1.251	(1.124 - 1.391)	<0.001	1.235	(1.144 - 1.334)	<0.001
08:00 – 11:59 *	1.000			1.000		
12:00 – 15:59	0.948	(0.886 - 1.014)	0.120	1.035	(0.992 - 1.080)	0.109
16:00 – 19:59	1.027	(0.956 - 1.102)	0.471	1.230	(1.165 - 1.300)	<0.001
20:00 – 23:59	1.087	(1.013 - 1.167)	0.021	1.406	(1.318 - 1.498)	<0.001
**Event Day**						
work day *	1.000			1.000		
non-working day	0.854	(0.778 - 0.937)	0.001	0.595	(0.562 - 0.630)	<0.001
**Event Time**						
00:00 – 03:59	0.487	(0.443 - 0.536)	<0.001	0.024	(0.020 - 0.029)	<0.001
04:00 – 07:59	0.521	(0.475 - 0.571)	<0.001	0.027	(0.022 - 0.034)	<0.001
08:00 – 11:59 *	1.000			1.000		
12:00 – 15:59	1.093	(1.017 - 1.175)	0.015	0.830	(0.730 - 0.943)	0.004
16:00 – 19:59	0.955	(0.892 - 1.023)	0.192	0.179	(0.147 - 0.219)	<0.001
20:00 – 23:59	0.904	(0.829 - 0.986)	0.023	0.081	(0.064 - 0.104)	<0.001
**Interaction: Admission Time x Admission Day**						
00:00 – 03:59 x non-working day	0.776	(0.667 - 0.903)	0.001	1.022	(0.939 - 1.111)	0.618
04:00 – 07:59 x non-working day	0.903	(0.780 - 1.045)	0.170	1.040	(0.963 - 1.123)	0.315
08:00 – 11:59 x non-working day *	1.000			1.000		
12:00 – 15:59 x non-working day	0.919	(0.835 - 1.013)	0.088	0.995	(0.936 - 1.058)	0.879
16:00 – 19:59 x non-working day	0.911	(0.818 - 1.015)	0.091	0.975	(0.910 - 1.044)	0.466
20:00 – 23:59 x non-working day	0.948	(0.847 - 1.062)	0.357	0.989	(0.913 - 1.070)	0.781
**Interaction: Event Time x Event Day**						
00:00 – 03:59 x non-working day	1.148	(0.982 - 1.341)	0.083	1.370	(1.143 - 1.642)	0.001
04:00 – 07:59 x non-working day	1.054	(0.920 - 1.208)	0.446	1.561	(1.404 - 1.736)	<0.001
08:00 – 11:59 x non-working day *	1.000			1.000		
12:00 – 15:59 x non-working day	0.981	(0.854 - 1.127)	0.790	0.961	(0.904 - 1.021)	0.197
16:00 – 19:59 x non-working day	1.055	(0.921 - 1.209)	0.438	1.409	(1.296 - 1.531)	<0.001
20:00 – 23:59 x non-working day	1.085	(0.961 - 1.224)	0.189	1.608	(1.457 - 1.774)	<0.001
